# Pericardial pseudocyst along atrioventricular groove

**DOI:** 10.1259/bjrcr.20200122

**Published:** 2020-12-18

**Authors:** Ajay Alex, Anoop Ayyappan, Jineesh Valakkada, Vivek V Pillai, Renjith Sreekantan, Rajalakshmi Poyuran

**Affiliations:** 1 Department of Imaging Sciences and Interventional Radiology, Sree Chitra Tirunal Institute for Medical Sciences and Technology, Trivandrum, India; 2 Department of Cardiovascular & Thoracic Surgery, Sree Chitra Tirunal Institute for Medical Sciences and Technology, Trivandrum, India; 3 Department of Pathology, Sree Chitra Tirunal Institute for Medical Sciences and Technology, Trivandrum, India

## Abstract

Cystic lesions in relation to the pericardium are a rare congenital lesion with an estimated incidence of 1 per 100,,000. Pericardial cysts may be classified as congenital or acquired. Here, we present a case of a pericardial pseudocyst having a horseshoe configuration along the atrioventricular groove in a middle-aged subject with no previous relevant medical history. The patient underwent open surgery for the same with histopathological diagnosis being established. This paper highlights the differentials for a cystic pericardial lesion in imaging in addition to the histopathological entity of a pericardial pseudocyst.

## Background

Cystic lesions adjacent to the pericardium are a rare congenital lesion altogether, with pericardial cyst being one of the commonest among them. Pericardial cysts in general have an estimated incidence of about 1 per 100,000.^1^ The aetiology of pericardial cysts are classified as congenital and acquired categories, with the congenital or idiopathic type being the more common.^2,3^ The majority of these cysts are congenital in origin. The incidence rates of acquired pericardial pseudocyst are not available in the literature. An inflammatory process is attributed to the formation of acquired pericardial cysts, which comprise pseudocysts as well as encapsulated and loculated pericardial effusions.^4^ It is even rarer for an insidiously detected cystic pericardial lesion to have haemorrhage within and being symptomatic with only a handful of case reports, most of which have a known prior medical or surgical history predisposing to the same.

Herein, we report a case of a heterogeneous cystic lesion almost wrapping the heart along the AV grooves and crux, with a unique horseshoe configuration in a patient in the absence of a prior medical or surgical history.

## Case report

**Figure 1.**
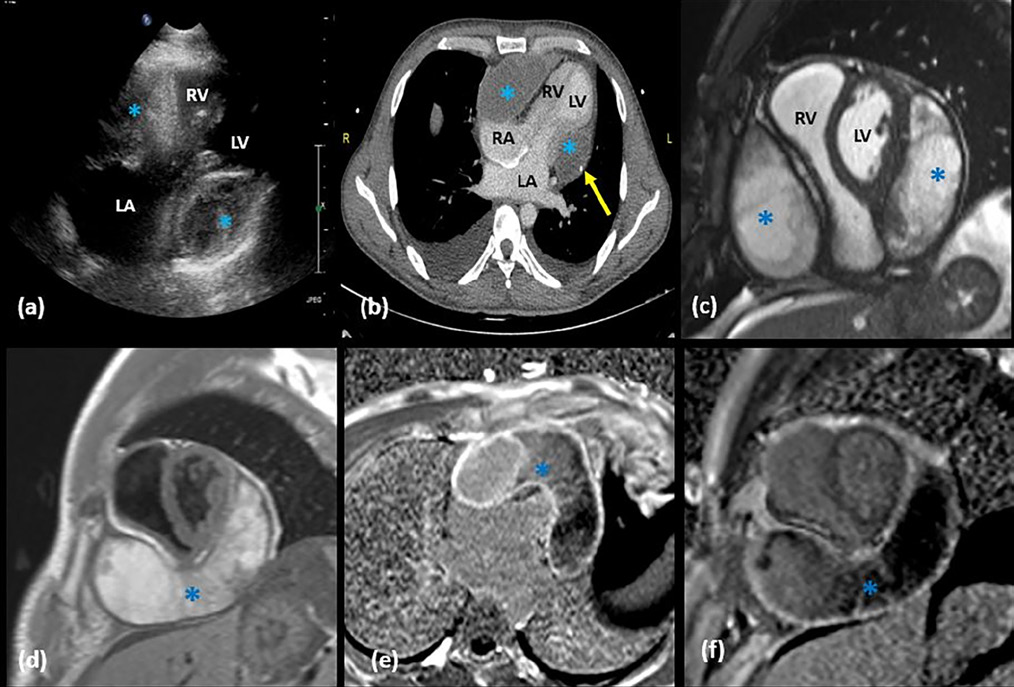
1(a) Echocardiogram (in IU 22, Philips) done in apical 4 CH chamber view shows a heterogeneous hyperechoic lesion (*) in the right and left AV groove region causing significant compression on the LV & RV chambers. 1 (b) Post contrast CT [in Brilliance ICT 256 slice, Philips Healthcare, Cleveland, OH] axial section showing hypodense cystic lesion (*) along the right and left AV groove with peripheral wall enhancement. A focus of calcification (yellow arrow) is noted in the thick wall towards the left side. In addition, bilateral pleural effusion secondary to heart failure is also seen. 1 (c–f) Cardiac MRI [in Avanto Fit 1.5T,Siemens, Erlangen, Germany)], SA images (c, d, f) and 4 CH image (e) showing the predominantly hyperintense thick walled cystic lesion (*) with horseshoe configuration (d–f) along the AV groove posteroinferior to the heart. T1 SA slice (d) showed hyperintense content indicative of haemorrhagic content. Post-contrast delayed PSIR image (e, f) showing the peripherally enhancing thick wall of the cyst. Otherwise, no significant pericardial effusion was detected in the present imaging. AV, atrioventricular; 4 CH, four chamber; LA, left atrium; LV, left ventricle; PSIR, phase sensitive inversion recovery; RA, right atrium; RV, right ventricle; SA, short axis.

34-year-old physically active male, presented with dyspnoea on exertion (NYHA Class II) for 4 months duration with associated pedal oedema. The medical history of the patient was unremarkable for any fever, chest pain, trauma, cough, pancreatitis, or weight loss. He was not a smoker or an alcoholic. Clinical evaluation revealed elevated JVP with loud P2 and wide S2 split. ECG did not show any changes of myocardial ischaemia nor cardiac enzyme elevation. Echocardiography revealed an extracardiac heteroechoic lesion causing significant compression on the cardiac chambers (Figure 1a). There were echogenic areas within the lesion with no definite vascularity in the Doppler assessment.

**Figure 2.**
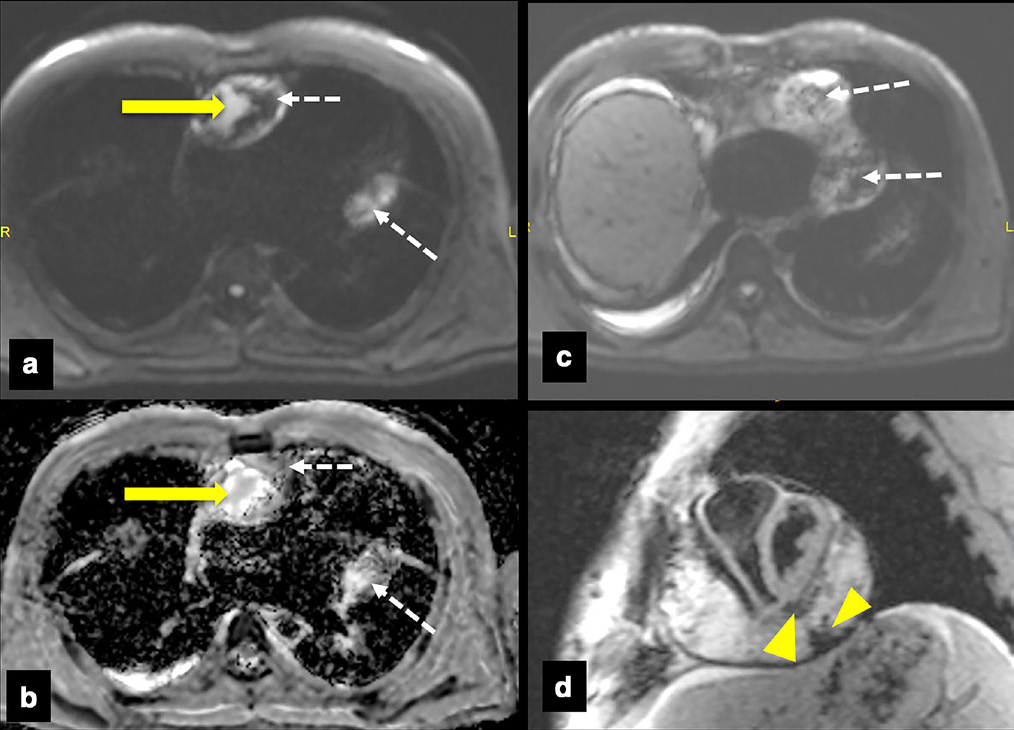
DW (a, c)and ADC (b) images of heart in axial section showing areas of diffusion restriction at b-value = 400 s mm^−2^, in right AV groove region (yellow arrows in a, b) and areas of non-restriction in other locations (Dotted arrows in a–c). GRE image (Figure 2 d) in short axis section showing areas of blooming with in the walls of the cyst (yellow arrowheads). ADC, apparent diffusion coefficient; DW, diffusion-weighted; GRE, gradient refocussed echo images.

The patient underwent CECT (Figure 1b) and cardiac MRI (Figure 1c–f) which revealed a heterogeneous, predominantly cystic lesion in between visceral and parietal layers of pericardium along the AV groove extending from the right to the left AV groove region along the inferior aspect of heart with longest dimension measuring 10 cm. The lesion showed T1 hyperintense contents within likely due to haemorrhage and separate non-enhancing areas in post-contrast T1 andT2 isointense areas suggestive of organised clots. There were mixed areas of diffusion restriction and non-restriction in diffusion-weighted sequences(Figure 2 a-c) with mild blooming in gradient sequences (Figure 2d)which can be explained by haemorrhagic contents inside. Diastolic motion restriction of ventricular myocardium was noted in MRI. ECG-gated CECT after giving 100 ml contrast at 3 ml s^−1^ and acquiring at 45 s delay in a 256 slice CT showed a tiny focus of peripheral calcification on the wall of the lesion. Peripheral cyst wall showed contrast enhancement, however not in the remaining pericardium or solid intracystic contents. Coronary arteries appeared normal adjacent to the cyst.

Due to the unique orientation of the lesion along the AV groove in the inferior aspect of heart, differentials considered were pericardial cyst with haemorrhage within, lymphangioma, hydatid disease, and a rare possibility of a duplication cyst. Image-guided aspiration was not recommended by the multidisciplinary team considering the complex location and extend of the lesion, chance of intracardiac injury, incomplete resolution of the cyst and dilemma with final diagnosis.

**Figure 3.**
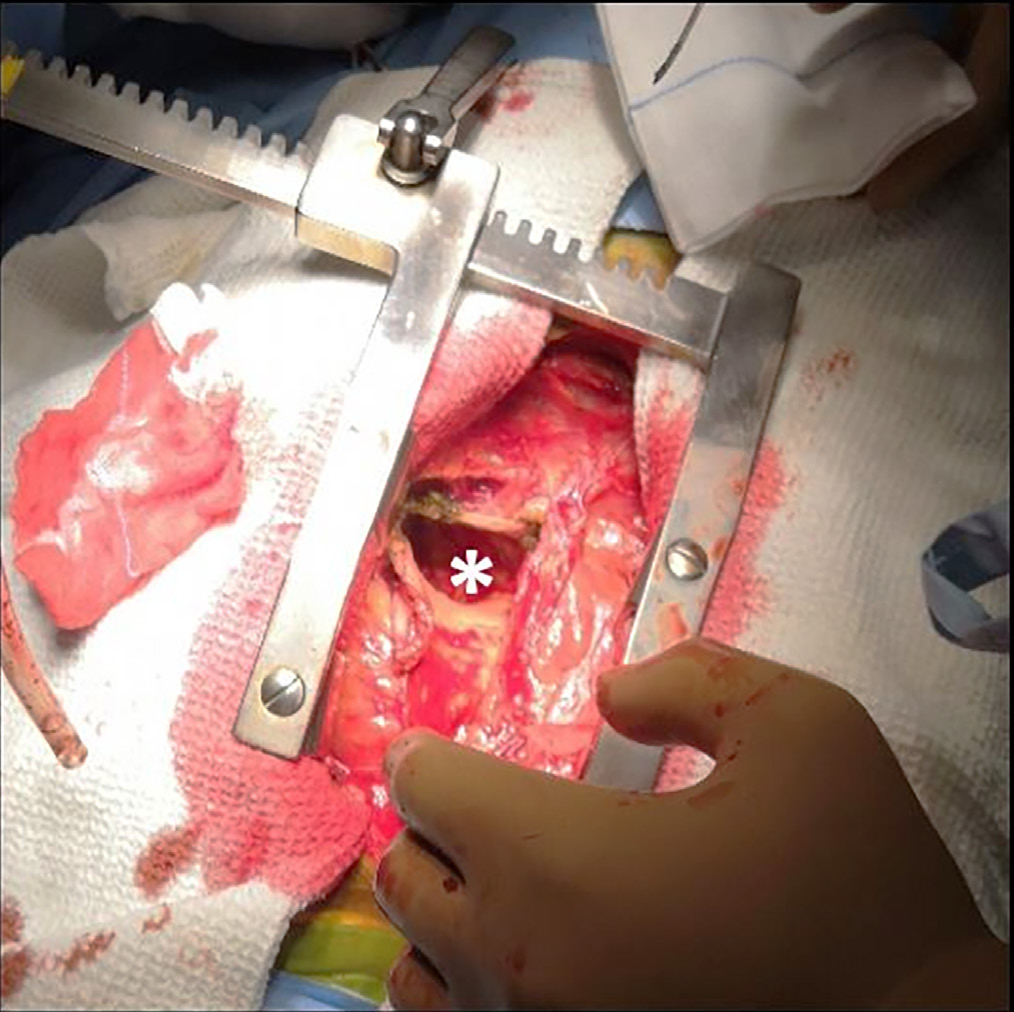
Intraoperative photograph showing the cyst cavity (*) after emptying of the contents with the thickened wall.

Significant compression on the cardiac chambers by the cyst and resulting right heart failure symptoms warranted a surgical de-roofing of the cyst after sternotomy. Intraoperatively (Figure 3) the cyst had a thick wall with horseshoe shape anterior to RA, inferior and anterior to RV, inferior and posterior to LV, and contained old clotted and altered blood. Histopathological samples obtained from the wall as well as of the fluid within the cyst were sent for microscopic examination and culture. Post-operative echocardiogram showed no residual lesion with mild thinning and dyskinesia at the inferior part of the septum. The patient was discharged 10 days after surgery.

**Figure 4.**
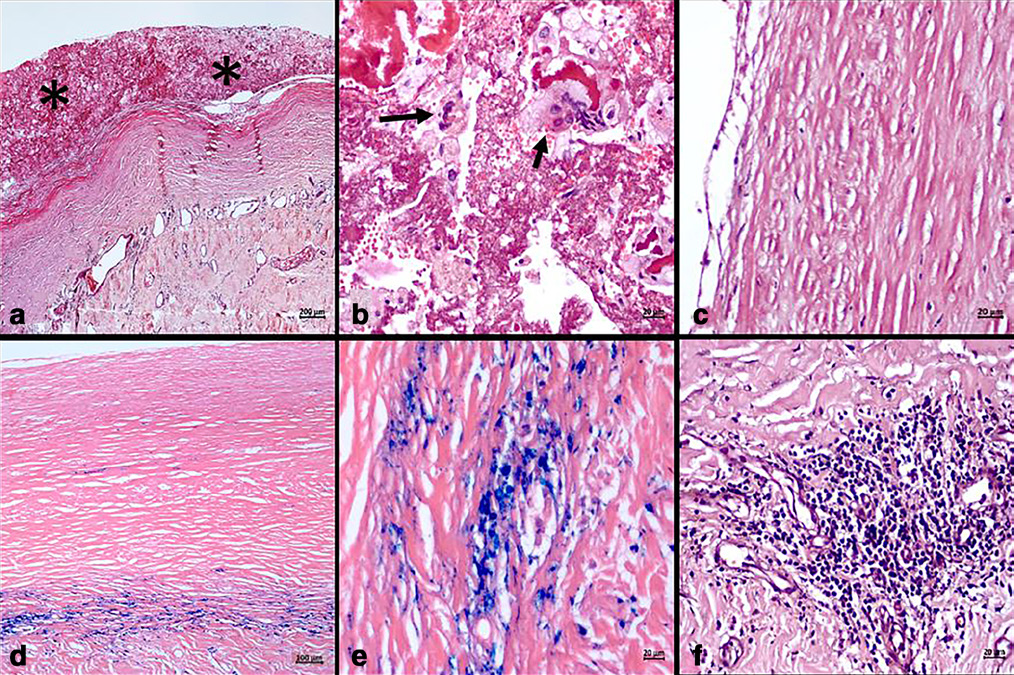
Thick fibrocollagenous cyst wall (a) and adherent fibrinous and haemorrhagic content (_*_) with foreign body type of multinucleate giant cells (B-arrow) and macrophages (b). The cyst wall lacks any lining epithelium (c) and shows hemosiderin pigment (d, e) and focal lymphocytic infiltration (f). [Stain: a–c, f: Haematoxylin and eosin; d, e: Perls Prussian blue; Magnification = Scale bar (a = 200 µm; d = 100 µm; b, c, e, f = 20 µm)]

Histopathological examination of the cyst wall revealed thickened fibrocollagenous tissue with adherent fibrin on the inner aspect containing hemosiderin pigment, macrophages, and few multinucleate giant cells. The cyst wall itself was devoid of any lining epithelium (Figure 4) and showed abundant hemosiderin pigment and focal lymphocytic infiltration. The cyst content was composed of fibrin and RBCs with macrophages and occasional multinucleate foreign body giant cells, some hemosiderin-laden. There was no evidence of granuloma, fungi, hydatid cyst, lymphangioma-like morphology, mesothelial cells, or any atypical cells. Immunohistochemistry with Pan-cytokeratin (PanCK) was negative confirming the lack of any epithelial cells. Overall, the features were of a pseudocyst with organising haemorrhage. Amylase and lipase evaluation of the fluid also revealed no abnormality. Samples were subjected to microbiological evaluation as well which revealed no evidence of infection including hydatid disease or granulomatous inflammation. Post-procedure, the patient had relief of his symptoms and was doing well.

## Discussion

Cystic pericardial lesions are relatively uncommon lesions in adults with the majority being detected incidentally (1). Common locations are the right cardiophrenic angle (51–70%), left cardiophrenic angle (28–38%), and rarely in the superior mediastinum (8–11%).^5–7^ Rarely pericardial cysts have been described in the right ventricle and interventricular septum.^8,9^ However, current literature shows no description of an extensive cystic lesion along both the AV grooves and inferior aspect of heart as in the present case.

Anatomically, the pericardium consists of an outer fibrous layer and an inner serous sac containing a parietal and visceral layer composed of mesothelial cells (1). Congenital pericardial cysts are due to abnormal fusion or lack of fusion of mesenchymal lacunae prenatally (2). Congenital cysts have a wall formed of connective tissue lined by mesothelial cells; however, the pseudocysts lack the same.^10^

Acquired pericardial cysts or pseudocysts are encapsulated or loculated pericardial effusions caused by inflammation (secondary to trauma, rheumatic pericarditis, previous surgery, or past myocardial infarction).^4,11,12^ A literature review revealed reports of hydatid disease and metastasis as other aetiologies of cystic pericardial lesions.^13–15^ Haemorrhage into a pericardial cyst is extremely rare with only a few reports only in literature with all reports having a significant medical or surgical history as a cause for the haemorrhage including trauma.^12,16,17^

Although the majority of the pericardial cysts don’t produce any symptoms (70% patients), compression by large cysts can instigate dyspnoea, pain, cough, palpitations, dysphagia, weight loss, and paroxysmal tachycardia.^18^

Main differential diagnoses, which should be considered when evaluating cystic pericardial lesions, include congenital pericardial cyst, localised pericardial effusion or pericardial pseudocyst, hydatid cyst, lymphangioma, cystic mediastinal teratoma, Morgagni hernia, neurenteric cyst, and congenital cysts of primitive foregut origin. Clinicians and radiologists should be aware of these differentials and decide on further work-up.^19^ In MRI, congenital pericardial cysts are generally well-defined lesions having a thin wall with contents showing homogenous T2 hyperintensity and no contrast enhancement, most commonly located along the right anterior cardiophrenic angle.^20^ Pericardial pseudocysts can have heterogeneous MR signals depending on the contents (though predominantly T2 hyperintense) with a thick enhancing wall and no enhancement of the contents of the lesion in post-contrast images. Hydatid cysts will have a multicystic appearance with the classic appearance of daughter cysts within a larger cyst. Lymphangiomas are identified as T2 hyperintense lesions with a multiloculated appearance and rarely by the presence of fluid–fluid levels secondary to haemorrhage in some of the locules.^21^ Teratomas have fat or calcium as contents in majority cases and appear as multilocular cysts.^22^ Morgagni hernia can be differentiated by demonstrating the defect in the diaphragm and the continuity of the contents into the abdomen. Neurenetric cysts have thick uniform wall with a typical location in posterior mediastinum posterior to heart with associated vertebral body defect and intraspinal extension.^23^ Generally, foregut duplications cysts have thick walls with the location being mainly remote from the pericardium.

**Figure 5.**
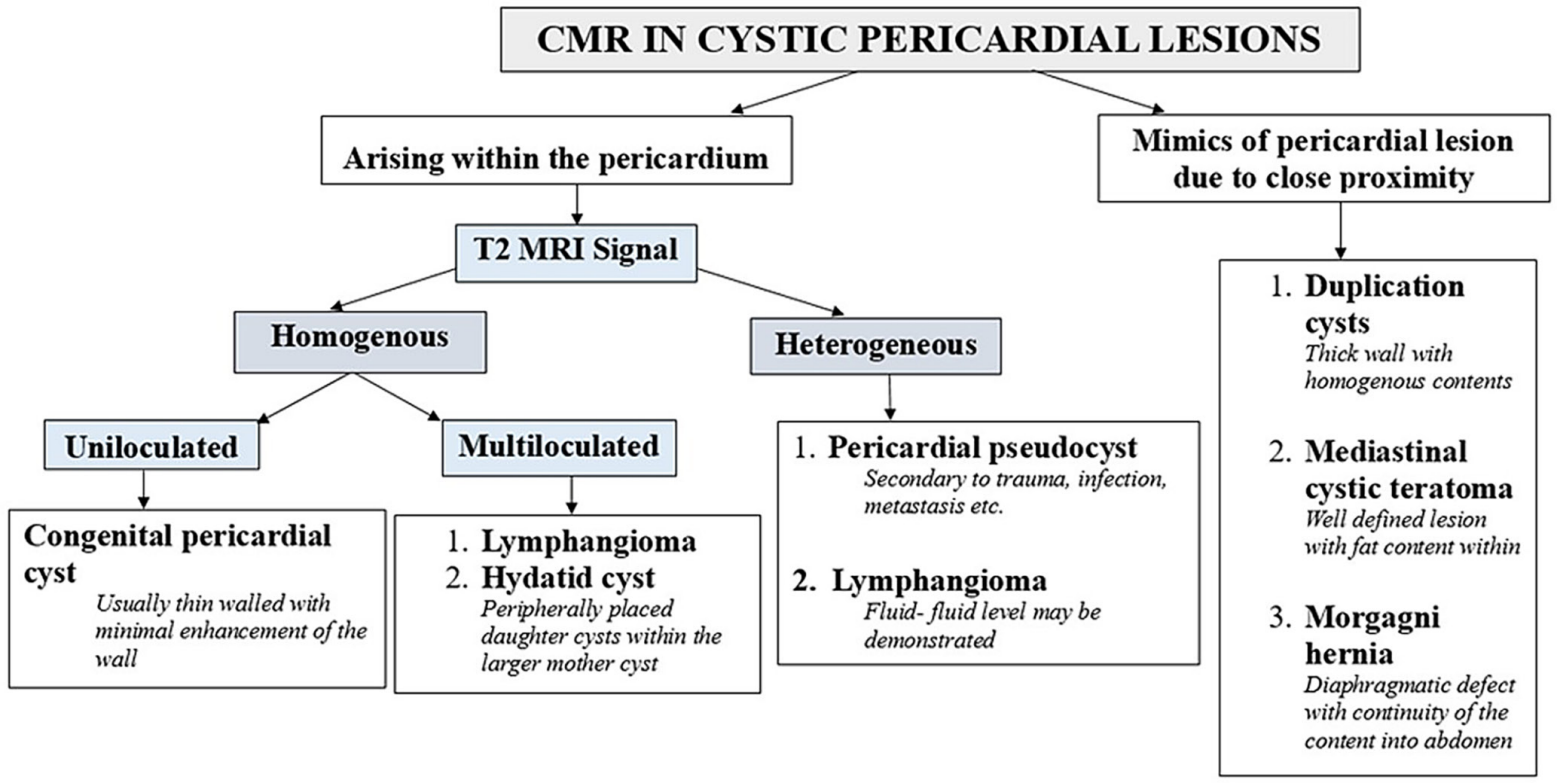
Algorithm for cardiac MRI diagnosis in pericardial cystic lesions. CMR, cardiac MR.

The American Society of Echocardiography (ASE) recommends cardiac CT or CMR if a pericardial cyst is suspected after preliminary X-ray or echocardiography.^24^ CMR is considered superior to CT in differentiating non-serous fluid cysts and cysts with haemorrhage from a solid mass, which can have high attenuation on CT.^1^ On contrast CMR, contents of the cysts are hyperintense in T2 with no post-contrast T1 enhancement. A plausible algorithm for diagnosis in cystic pericardial lesions using cardiac MR is summarised in Figure 5.

Management of cystic lesions depends on the nature of the lesion on imaging and clinical symptoms. The management of congenital pericardial cysts and pseudocysts may be conservative or surgical. Indications for the surgical resection are symptomatic cases, large size, and uncertain malignant potential to prevent complications.^24^ Treatment options include percutaneous aspiration, ethanol-induced sclerosis, and resection through video-assisted thoracoscopic surgery, resection via thoracotomy, median sternotomy, or laparoscopic approach.^8^

## Learning points

1. Pericardial cyst can occur in inferior AV groove apart from lymphangioma and duplication cysts.
2. Haemorrhage in a pericardial cyst can be mistaken for an aggressive lesion by echocardiogram but easily identified using MRI.
3. Mass effect by the cyst is an indication for surgical management.

## Conclusion

This case report highlights the existence of a pericardial pseudocyst as an entity and to be kept in the possible differentials for the atypical appearance of pericardial cystic lesions. Also, the fact that pericardial cysts may have haemorrhagic content with unusual appearance encompassing varied locations and configurations even in cases with no documented prior medical or surgical history, proving it to be an imaging challenge in reaching a diagnosis. The explanation for this unexpected location and configuration of the pericardial cyst is uncertain, however maybe due to the underlying haemorrhage or inflammation.
